# Atomic, electronic and magnetic structure of an oxygen interstitial in neutron-irradiated Al_2_O_3_ single crystals

**DOI:** 10.1038/s41598-020-72958-9

**Published:** 2020-09-28

**Authors:** V. Seeman, A. Lushchik, E. Shablonin, G. Prieditis, D. Gryaznov, A. Platonenko, E. A. Kotomin, A. I. Popov

**Affiliations:** 1 Institute of Physics, University of Tartu, W. Ostwald Str. 1, 50411 Tartu, Estonia; 2 Institute of Solid State Physics, University of Latvia, Kengaraga 8, Riga, 1063 Latvia; 3 Max Planck Institute for Solid State Research, Heisenbergstr. 1, 70569 Stuttgart, Germany; 4 Institute Laue Langevin, 6 Rue Jule Horovitz, 38042 Grenoble, France

**Keywords:** Materials science, Physics

## Abstract

A single radiation-induced superoxide ion $O_{2}^{ - }$ has been observed for the first time in metal oxides. This structural defect has been revealed in fast-neutron-irradiated (6.9×10^18^ *n*/cm^2^) corundum (α-Al_2_O_3_) single crystals using the EPR method. Based on the angular dependence of the EPR lines at the magnetic field rotation in different planes and the determined *g* tensor components, it is shown that this hole-type $O_{2}^{ - }$ center *(i)* incorporates one regular and one interstitial oxygen atoms being stabilized by a trapped hole (*S* = 1/2), *(ii)* occupies one oxygen site in the (0001) plane being oriented along the *a* axis, and *(iii)* does not contain any other imperfection/defect in its immediate vicinity. The thermal stepwise annealing (observed via the EPR signal and corresponding optical absorption bands) of the $O_{2}^{ - }$ centers, caused by their destruction with release of a mobile ion (tentatively the oxygen ion with the formal charge −1), occurs at 500–750 K, simultaneously with the partial decay of single *F*-type centers (mostly with the EPR-active *F*^+^ centers). The obtained experimental results are in line with the superoxide defect configurations obtained via density functional theory (DFT) calculations employing the hybrid B3PW exchange-correlation functional. In particular, the DFT calculations confirm the $O_{2}^{ - }$ center spin *S* = 1/2, its orientation along the *a* axis. The $O_{2}^{ - }$ center is characterized by a short O–O bond length of 1.34 Å and different atomic charges and magnetic moments of the two oxygens. We emphasize the important role of atomic charges and magnetic moments analysis in order to identify the ground state configuration.

## Introduction

Aluminium oxide possesses various fascinating properties allowing to use corundum (α-Al_2_O_3_) for different applications in science and technology. Because of high tolerance against heavy irradiation, low swelling even under high fast neutron fluences and ability to maintain mechanical and electric integrity, α-Al_2_O_3_ single crystals and transparent polycrystalline ceramics are widely used for fission-based energetics and are considered as promising diagnostics/window materials in further deuterium-tritium fusion devices (see, e.g.^1–3^ and references therein).

It is generally accepted that radiation resistance of functional materials is mainly determined by the accumulation and aggregation of primary structural defects—vacancy-interstitial Frenkel pairs. In radiation-sensitive alkali halide crystals, the formation energy of a Frenkel pair is notably less than the energy gap value, *E*_FP_ < *E*_g_ and the efficient creation of Frenkel defects occurs via the decay of self-trapping anion excitons or the recombination of relaxed electron-hole pairs^4,5^. In rare gas crystals (cryocrystals), the situation is similar and self-trapped excitons are considered to be responsible for radiation damage as well (see, e.g.^6,7^). On the other hand, these ionization (non-impact) mechanisms of defect creation are not realized in wide-gap metal oxides with opposite energetic inequality, *E*_FP_ > *E*_g_ (the value of *E*_FP_ is up to 2-3 times higher). Therefore, the elastic collisions of incident energetic particles (neutrons, ions, electrons) with atoms/nuclei of materials are mainly responsible for radiation damage in radiation-resistant metal oxides. Note that such displacement (collision, impact) mechanism almost completely describes the creation of Frenkel defects under fast neutron irradiation^8,9^. At the same time, ~GeV swift heavy ions provide extremely high density of electronic excitations within cylindrical tracks (>20 keV/nm), and, as a result, additional contribution of ionization losses to radiation damage of metal oxides is expected as well (see, e.g.^10,11^and references therein).

Radiation damage of α-Al_2_O_3_ caused by fast neutrons, energetic electrons and ions have been investigated for many years^1,2,9,12–23^. The optical characteristics of the anion-vacancy-related elementary Frenkel defects—*F*^+^ and *F* centers (one or two electrons trapped by an oxygen vacancy) have been thoroughly studied in irradiated and additively colored Al_2_O_3_ crystals^13–16^. In addition, the EPR signal related to the paramagnetic *F*^+^ centers has been also revealed in neutron-irradiated α-Al_2_O_3_ single crystals^24^. The thermal annealing of the absorption bands related to *F*-type single centers in additively colored corundum takes place above 1300 K, i.e. significantly higher than that in the irradiated crystals, where still immobile *F*^+^ and *F* centers recombine with becoming mobile oxygen interstitials^14,15,17–19^.

Nevertheless, the complementary Frenkel defect to the *F*-type center—a single interstitial oxygen—still remains the most hidden primary defect in α-Al_2_O_3_ and other wide-gap metal oxides. The main efforts to find experimental manifestations of radiation-induced oxygen interstitials have been undertaken in MgO single crystals (see, e.g. ^25–28^ and references therein). Halliburton and Kappers^25^ discovered the EPR signal of an oxygen interstitial in the form of a quasi-molecule (superoxide) $O_{2}^{ - }$ and interpreted this center with spin *S* = 1/2 as an analog of an *H* center in alkali halides (an interstitial halogen atom or $X_{2}^{ - }$ dihalide molecule occupying a single anion site^29^). However, an analogy was not complete because the $O_{2}^{ - }$ center, detected in neutron-irradiated MgO and the paramagnetic nature of which had been a trapped hole, was associated with a cation vacancy located nearby $O_{2}^{ - }$ (actually three slightly different *H*-type centers were detected in Ref.^25^, see also Table 1). Up to now, a single $O_{2}^{ - }$ center located in a regular lattice region (i.e. not associated with any other defect) was not detected in MgO, Al_2_O_3_ and other metal oxide crystals. At the same time, a family of paramagnetic $O_{2}^{ - }$-type centers with superoxide ions adjacent to some other structural defect/impurity have been revealed by the EPR method in the bulk and/or at the surfaces of MgO^25,27,30^, CaO^31–33^, SrO^34–36^, ZnO^30^, Na-β-Al_2_O_3_ crystals^37^ and other oxide materials. It should be mentioned that the EPR signal of a single $O_{2}^{ - }$ molecule was also detected in a pure SrO crystal^35^. However, this molecular center was detected in an X-ray irradiated nonstoichiometric crystal possessing oxygen excess and its formation should be considered as a reversible hole trapping at an as-grown defect, rather than at a radiation-induced one.

**Table 1:** EPR parameters for the $O_{2}^{ - }$ centers in the bulk (*B*) or at the surface (*S*) of different metal oxides.

*host*	*g*_x_	*g*_y_	*g*_z_	Ref.
α-Al_2_O_3_ (*B*)	2.009(6)	2.004(9)	2.045(9)	
Na-β-Al_2_O_3_ (*B*)	2.0079	2.0023	2.0591	^37^
MgO (*B*)	2.0069^*^	2.0017^*^	2.0776	^25^
MgO (*B*)	2.0073^*^	2.0017^*^	2.0770	^25^
MgO (*B*)	2.0077^*^	2.0020^*^	2.0662	^25^
MgO (*B*)	2.0059	2.0011	2.0767	^27^
MgO (*B*)	2.0061	2.0011	2.0761	^27^
MgO (*S*)	2.0073^*^	2.0011^*^	2.077^*^	^30^
CaO (*B*)	2.0064^*^	2.0004^*^	2.0931^*^	^31^
CaO (*B*)	2.0084^*^	2.0013^*^	2.0890^*^	^31^
CaO (*S*)	2.0059	2.0004	2.093	^32^
CaO (*S*)	2.0067	2.0015	2.089	^33^
SrO (*B*)	1.9957^*^	1.9914^*^	2.1934	^35,36^
SrO (*S*)	2.006	2.001	2.102	^34^
ZnO (*S*)	2.0082^*^	2.0020^*^	2.051*	^30^

The values^*^ marked with asterisk are rearranged with respect to the axes notation used in the present study.

According to theoretical calculations, single oxygen interstitials in the neutral charge state in MgO and Al_2_O_3_ are unstable with respect to transformation into a dumbbell configuration, their atomic charges and diffusion in MgO and Al_2_O_3_ crystals have been theoretically analyzed^38–41^. Diffusion of charged oxygen interstitial defects was also discussed in Ref.^42^. Note that the EPR-active trapped-hole centers in alkaline earth and aluminium oxides are so-called *V*-type defects that consist of a hole trapped at a regular oxygen (an $O^{ - }$ ion ) nearby some structural defect (predominantly a cation vacancy) or/and an impurity ion (see, e.g.^43–50^ and references therein). Also, a neutron-irradiated magnesium aluminate spinel (MgAl_2_O_4_) demonstrated the presence of *V*-type defects, which was confirmed by experimental EPR studies^51–53^ and following theoretical considerations^54^. The latter theoretical study was the first attempt of hyperfine coupling constants calculations using the first-principles methods for MgAl_2_O_4_ spinel, and to the best of our knowledge, other oxides. Notice that the first-principles calculations, see^40–42,54^, were performed with the hybrid exchange-correlation functionals.

The present study reports, for the first time, the EPR study of single oxygen interstitials in a form of $O_{2}^{ - }$ superoxide ions created in α-Al_2_O_3_ single crystals under fast neutron irradiation. A special attention is paid to the comparative investigation and analysis of the thermal annealing of the EPR signals of $O_{2}^{ - }$ and *F*^+^ centers and the corresponding absorption bands induced by fast fission neutrons. This is a logical continuation of our study on the *V*-type defects in neutron irradiated MgAl_2_O_4_ combining the EPR, optical methods ^51–53^and DFT calculations therein^54^.

## Experimental

Nominally pure single crystals of α-Al_2_O_3_ were grown using the Czochralski method by Union Carbide Corporation. The crystals were irradiated by fission neutrons with energy exceeding 0.1 MeV and cumulative fluence of about 6.9×10^18^ *n*/cm^2^ at the Oak Ridge National Laboratory. Because of permanent cooling, the sample temperature during irradiation did not exceed 60 °C. Irradiation with X-rays was performed using a tungsten tube operated at 36 kV and 15 mA.

The EPR measurements were made with an X-band (9.8 GHz) spectrometer Bruker ELEXYS-II E500. The main measurements were performed at room temperature (RT), while the temperature dependence (from RT down to 5 K) of the EPR line width (peak-to-peak distance for derivative curves presented as ordinates in EPR figures) for the discovered defects was studied as well. In order to analyze the angular dependences of the EPR spectra, three irradiated samples aligned with different orthogonal crystallographic directions were used. The angular dependences were measured by the crystal rotation with a 2 or 5 degree step around the [0001] (further labeled as the *c* axis), $[1\overline{2}10]$ (*a* axis) or $[10\overline{1}0]$ (will be designated as *b*) axes, each of which was perpendicular to the direction of the magnetic field **B**. Note that the *b* axis is perpendicular to both the main crystal *c* axis and the *a* axis which is lying along the bisectrix of oxygen triangles. Varying microwave power was applied to separate the overlapping EPR spectra related to different paramagnetic centers. In particular, the spectrum of the *F*^+^ centers is clearly detectable at *P* = 0.05–0.5 mW (an optimal value of 0.1 mW), while higher *P* values are preferable for other paramagnetic centers. The program SpinFit (Bruker) was used to model/analyze the EPR spectra of the revealed centers, while a special Bruker program was applied for determination of their concentration in the irradiated samples.

The spectra of optical absorption at 1.5–6.5 eV were measured by a high-absorbance spectrometer JASCO V-660 with a double monochromator, while measurements in near vacuum ultraviolet (VUV, up to 8.5 eV) were performed by means of a vacuum monochromator VMR-2 and the hydrogen discharge light source. The difference between two absorption spectra sequentially measured at RT for the virgin crystal and the sample irradiated with fast fission neutrons was regarded as radiation-induced optical absorption (RIOA). In order to stay within experimental limits of optical density values (OD ≤ 4.0), a 0.09 mm thickness sample (about 8 × 8 mm^2^ plate, both sides polished and parallel to the main *c* crystal axes) was used. The RIOA spectra were decomposed into Gaussian components connected with the creation of different radiation-induced structural defects.

To analyze the recovery of radiation damage in Al_2_O_3_ single crystals, the following stepwise annealing procedure was applied: *(i)* the irradiated crystal was placed into a quartz reactor; *(ii)* heated to a certain temperature *T*_*pr*_ in a flowing atmosphere of highly pure argon; *(iii)* kept at this fixed *T*_*pr*_ for about 10 min; and, finally, *(iv)* cooled down by moving the reactor out of the furnace. Such multiple “heating-cooling-measuring” cycles were performed under the same conditions with the consistent rise of *T*_*p*r_ by 20 to 40 K. All the RIOA and EPR spectra detected during thermal annealing were measured at the same temperature—RT. The similar procedure was used in our recent studies devoted to fast-neutron-induced damage in MgAl_2_O_4_ single crystals^51,52^.

## Theoretical

The formalism of linear combination of atomic orbitals (LCAO) combined with the hybrid B3PW exchange-correlation functional was employed as implemented into the CRYSTAL17 computer code^55^_._ The basis sets for O and Al were adapted from our previous studies on α-Al_2_O_3_^41,42^. The all-electron basis set of atomic Gaussian type functions in the form 6*s*-2111*sp*-1*d* for O and the effective core pseudopotential with basis set functions of the 3*s*^2^3*p*^1^ external shell for Al. α-Al_2_O_3_ crystallizes in the rhombohedral structure (space group 167, $R\overline{3}c$) with 10 atoms in the primitive unit cell and 30 atoms in the conventional unit cell. The calculations of interstitial oxygens are performed in the supercell model using the transformation matrix (2 0 0, 0 2 0, 0 0 1) of the conventional unit cell comprising 120 atoms plus an additional interstitial oxygen atom^41^. The reciprocal space integration was performed by sampling the Brillouin zone with a 4 × 4 × 4 Monkhorst-Pack mesh for the supercell. The tolerances for the Coulomb and exchange integrals were 6, 6, 6, 6, 12 and the self-consistent field convergence on the total energy 10^6^ a.u. The calculations were spin polarized, and the charged supercells were calculated with a homogeneous neutralizing charge background.

The chosen calculation parameters allow an accurate description of basic bulk properties of α-Al_2_O_3_ (Table SI1 in the supplementary information to our previous paper^41^). The most relevant for the present study are the lattice parameters (*a* = 4.76 Å, *b* = 13.00 Å vs. *a* = 4.76 Å, *b* = 12.99 Å^56^ in the hexagonal setting) and band gap value (calculated values of 8.72 eV is slightly smaller than the experimental one of 9.4 eV^57^). The fractional coordinates of non-equivalent atoms are Al: (0, 0, 0.35) and O: (0.69, 0, 0.25) in the hexagonal setting. Four oxidation states (*OS*s) are usually considered for $O_{2}^{OS}$ species with *OS* = 2−, 1−, 0, 1+^58^. One of these oxidation states, namely *OS* = 1− (superoxide), plus an additional *OS* = 3−, represent paramagnetic defects and lead to formation of dumbbell configurations, and are of particular interest for the present experiments. The *OS* = 2− (peroxide) is also treated in the present study for a consistence. The *OS* = 3− was modeled by adding an extra electron to the supercell, which gives a ‘pseudo-dumbbell’ configuration^42^ due to symmetric (equal) atomic charges and magnetic moments of both oxygens despite a long 1.87 Å distance between them. The *OS* = 2− leads to formation of the dumbbell (a split interstitial) configuration with the shorter bond length of 1.44 Å^40,41^. We present a comparison of their formation energies, which represent the main characteristics of their stability in α-Al_2_O_3_. We carefully analyze the calculated properties of the *OS* = 1− serving as a model for a novel hole defect studied in the present experiments. The calculations of $O_{2}^{OS}$ in different *OS*s require use of the charged supercells. In order to avoid confusion between the defect (supercell) charge state (*q*) and *OS* we use following notations: $O_{2}^{2 - }$ (*OS* = 2−, *q* = 0), $O_{2}^{ - }$ (*OS* = 1−, *q* = +1) and $O_{2}^{3 - }$ (*OS* = 3−, *q* = −1).

The formation energy of a defect in the supercell in the charge state *q* was calculated in accordance with:

1 $$E_{F}^{q} = E^{q} - E^{Bulk} + E_{corr}^{q} - \mathop \sum \limits_{i} n_{i} \mu_{i} + q\left( {_{F} +_{VBM} + V} \right),$$

where $E^{q}$ and $E^{Bulk}$ represent the total electronic energies of supercells with a defect with the charge *q* and without a defect, respectively. In the present study, we rely on the first-order Makov-Payne scheme for $E_{corr}^{q}$ finite-size correction which is consistent with the literature data^59^. The calculation of $E_{F}^{q}$ needs the chemical potential $\mu_{i}$ of $n_{i}$ species removed or added. Thus, $n_{i}$ = 1 for the calculation of interstitial oxygen whereas $\mu_{i}$ equals one-half of the total electronic energy of *O*_2_ molecule (*OS* = 0) in the triplet state. $_{F}$ and $_{VBM}$ the Fermi energy and valence band maximum (VBM), respectively. $V$ is a correction for the average electrostatic potential alignment between the supercells with and without a defect.

## EPR spectra related to oxygen interstitials

Some of structural defects formed in heavily irradiated corundum single crystals possess paramagnetic origin and, as a result, can be investigated by the EPR method. In a fast neutron irradiated α-Al_2_O_3_ single crystal, a novel paramagnetic trapped-hole defect with *S* =1/2 and EPR spectrum at 342–352 mT has been revealed for the first time. In our opinion, this center could be ascribed to radiation-induced oxygen interstitial, which associates with a regular $O^{2 - }$ anion and forms a molecular ion located at a single anion site with a trapped hole—an $O_{2}^{ - }$ superoxide ion (hereafter $O_{2}^{ - }$ center). The experimental and theoretical facts that had enabled us to draw such conclusion are given below.

**Figure 1:**
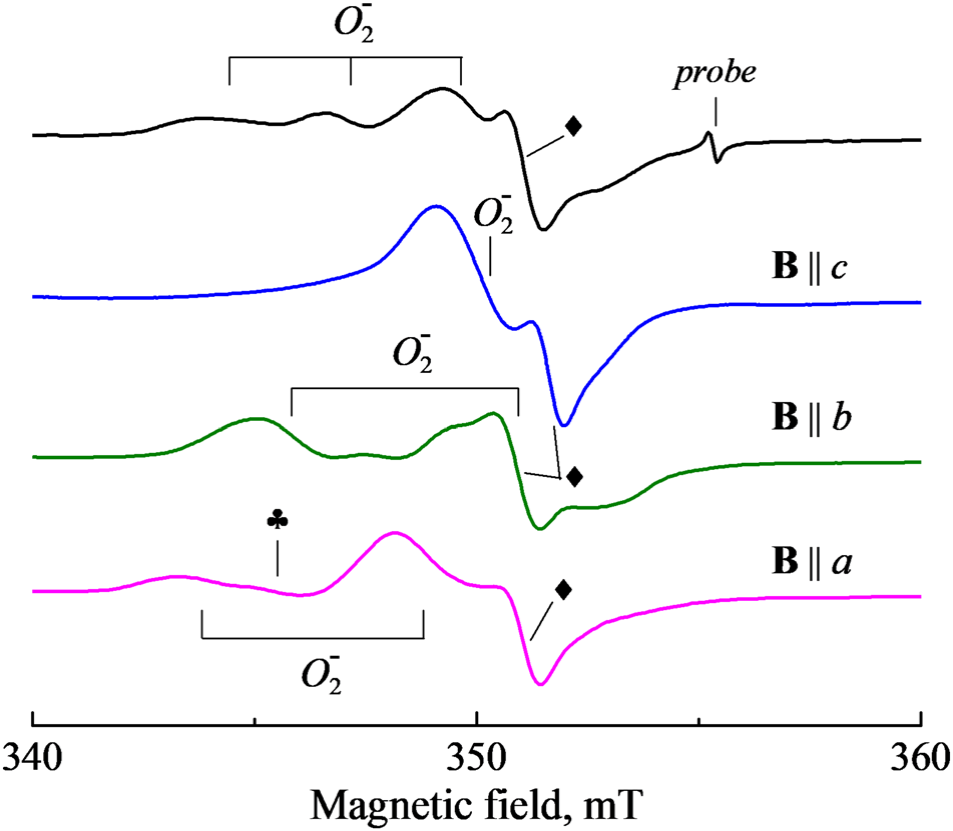
EPR spectra measured at different orientation of **B** with respect to *a*, *b* and *c* axes of a neutron-irradiated α-Al_2_O_3_ crystal. The upper spectrum corresponds to φ = 16° between **B** and the axis *a* in the (0001) plane. Symbols (♣ and ♦) mark EPR signals of two unidentified radiation defects, signal of a test sample corresponds to *g* = 1.9800. *T*= 295K, *P* = 0.6mW.

**Figure 2:**
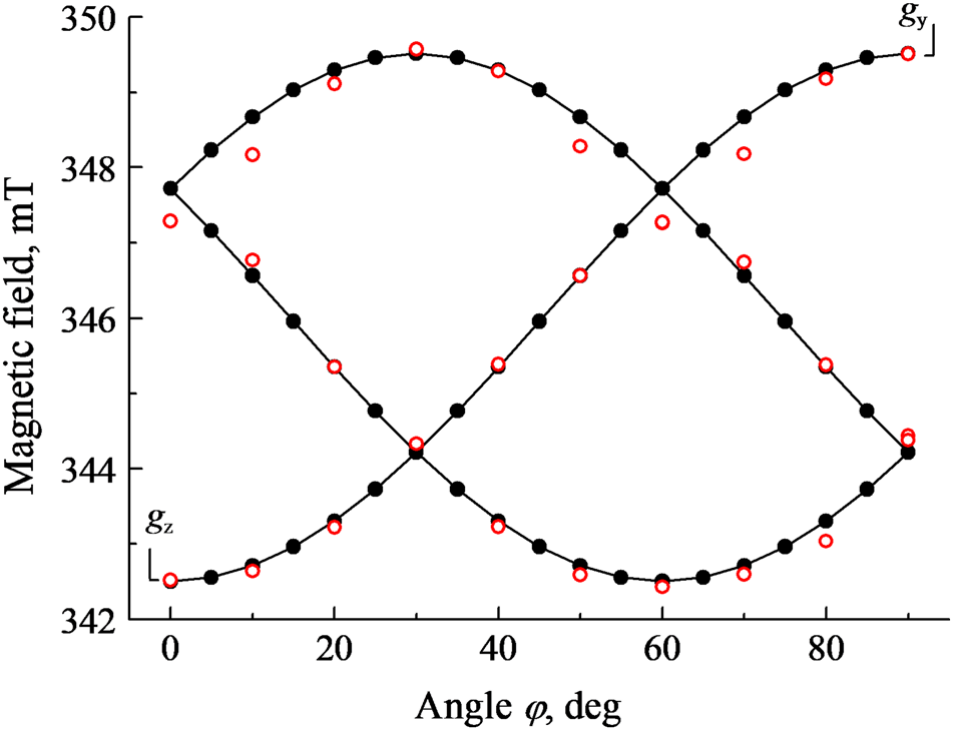
The angular dependences of the ${O}_{2}^{-}$ EPR lines at the **B** rotation in the (0001) plane in a neutron-irradiated α-Al_2_O_3_ crystal. The angle *φ* is measured between the direction of **B** and the axis *a*. Solid lines and filled circles− calculations, open circles − experimental points.

Figure 1 presents the EPR spectra of a neutron-irradiated α-Al_2_O_3_ crystal measured at the microwave power *P* = 0.6 mW for the case of an arbitrary orientation of the magnetic field in the (0001) crystal plane (the upper spectrum) as well as for **B** orientations along the *a*, *b* and *c* axes of the sample. Note that the upper spectrum contains three EPR lines with comparable peak intensities (better pronounced via EPR angular dependences, see Fig. 2) and a width of 1.7–1.5 mT, while the spectra observed at a certain **B** orientation consist of only two (**B**||*a* or **B**||*b*) or even one of these EPR lines (**B**||*c*). The above-mentioned EPR lines, ascribed to the $O_{2}^{ - }$ center, overlap partially with each other as well as with the lines associated with yet unidentified defects, which are marked by symbols (♣ and ♦) in Fig. 1. The presence of two other radiation-induced defects complicates the analysis of the EPR spectra.

Angular variations of the $O_{2}^{ - }$ line positions at the magnetic field **B** rotation in the crystallographic plane (0001) are shown in Fig. 2. At random **B** orientation in the (0001) plane, three EPR lines of comparable peak intensity can be clearly observed. Two of these lines are overlapped in the orientations **B**||*a* and **B**||*b*, the lines reach extremum positions (at *g*_z_ = 2.0459 and *g*_y_ = 2.0049) if **B** coincides with the *a* and *b* axis, respectively. Note that in case of **B**||*c*, only one line (*g*_x_ = 2.0096) is due to the $O_{2}^{ - }$ center. Such situation is illustrated in Fig. 1, while the case is not observed at angular dependences from Fig. 2.

**Figure 3:**
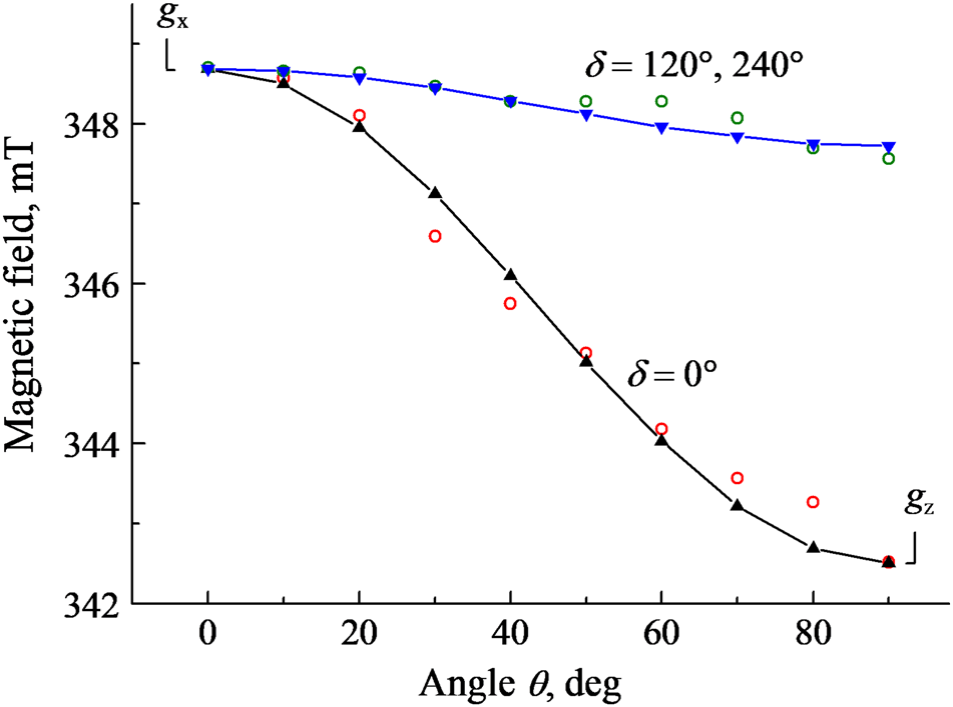
The angular dependences of the ${O}_{2}^{-}$ EPR line positions at the **B** rotation in the $(10\overline{1}0)$ plane (around *b* axis) in a neutron-irradiated α-Al_2_O_3_ crystal. The angle *Θ* is measured between the direction of **B** and the axis *c*. Solid lines and filled symbols—calculations, open circles—experimental points. The angle δ is between z axis of the ${O}_{2}^{-}$ and the crystallographic direction $[1\overline{2}10]$ (one of *a* axes).

When **B** is rotated in the $(10\overline{1}0)$ plane (around *b* axis), this single line at *g*_x_ = 2.0096 splits into two components as **B** is turned away from the **B**||*c* orientation. The angular variation (experimental points and calculations) presented in Figure 3 just corresponds to **B** rotation around *b* axis in the $(10\overline{1}0)$ plane; the angle *Θ* is measured between the **B** and axis *c*, while *δ* is the angle between *z* axis of the $O_{2}^{ - }$ and the crystallographic direction $[1\overline{2}10]$ (one of *a* axes). Two EPR lines shift to lower fields and reach extrema (*g*_z_ = 2.0459 and *g* = 2.016, respectively) at the orientation *Θ* = 90°*.* The analysis of angular dependences at the **B** rotation around *a* axis turned out to be hampered due to the partial overlapping with the EPR lines related to other defects and are not presented in the paper.

The above-mentioned facts demonstrate that the *z* and *y* axes of the *g* tensor both lie in the plane (0001), whereas the *z* axis is parallel to the *a* crystal axis and *x*||*c*. The principal values of the *g* tensor are determined as follows: *g*_z_ = 2.0459, *g*_y_ = 2.0049 and *g*_x_ = 2.0096. Based on the analysis of angular dependences of the EPR spectra, the orientation of the paramagnetic $O_{2}^{ - }$ center is determined to be along the *a* axis. The last circumstance indicates that the molecular axis of this paramagnetic center is not declined because of influence of any other structural imperfection/defect in the immediate vicinity of the $O_{2}^{ - }$. As a result, we are dealing with a single superoxide ion that will be also confirmed by the density functional calculations (see Sect. 6).

**Figure 4:**
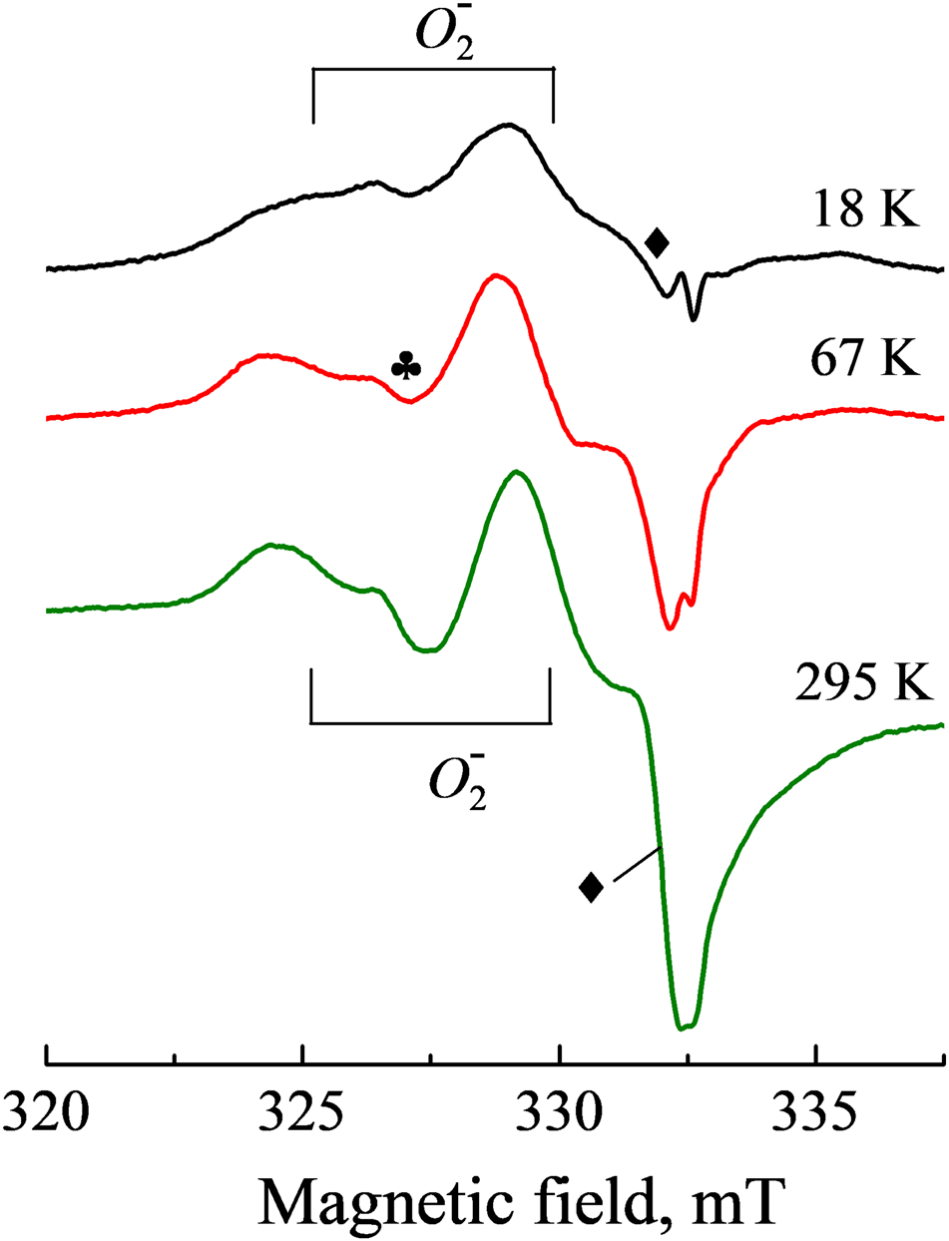
The EPR spectra of the ${O}_{2}^{-}$ center measured at different temperatures in a neutron-irradiated α-Al_2_O_3_ crystal. **B**||*a, P* = 0.063 mW for RT and *P* = 0.02 mW for 67 and 18 K.

Note that there is no recorded hyperfine structure (HFS) in the EPR spectra of the $O_{2}^{ - }$ centers presented in Fig. 1. In general, the absence of HFS can be explained by insufficient resolution due to the spectra detection at RT. However, temperature decrease down to 5 K does not improve resolution of the EPR lines (see Fig. 4). In addition, there was detected an obvious saturation of the $O_{2}^{ - }$ center signal at low temperatures that becomes especially strong at *T* ≤ 10 K. Therefore, most of the EPR measurements were carried out at RT.

The following additional arguments support the model of the revealed paramagnetic center as the $O_{2}^{ - }$ molecular ion. Firstly, a positive shift of the *g* tensor components relative to the *g*_e_ = 2.0023 for the free electron confirms “a hole origin” of the center with *S* =1/2. Secondly, based on the literature data presented in Table 1, the certain ratio between the *g* tensor components is typical for the $O_{2}^{ - }$ molecules (associated with some other imperfection) already detected in different metal oxides. More specifically, the values of two components are close and significantly lower than that for the third *g* tensor component, *g*_z _> *g*_y _≈ *g*_x_ . Moreover, the *g*_z_, *g*_y_ and *g*_x_ values for the $O_{2}^{ - }$ center detected in our neutron-irradiated α-Al_2_O_3_ (see the top-most table row) and for the “associated” $O_{2}^{ - }$ molecules in the bulk or at the surface of different metal oxides (Na-β-Al_2_O_3_, MgO, CaO, SrO, ZnO) are fairly close to one another (see Table 1).

Furthermore, the experimentally determined *g* values and center orientation as well as a rather high thermal stability of the revealed $O_{2}^{ - }$ centers (see next Sect. 5) do not match the corresponding parameters of the *V*-type hole centers (a hole trapped at a regular $O^{2 - }$ ion next to an aluminium vacancy (*V*_*Al*_), i.e. $O^{ - } V_{Al}$, which could also be formed under neutron irradiation in α-Al_2_O_3_. The results presented in Ref.^44^ clearly support the production of *V*_*Al*_ under irradiation of corundum by fast neutrons. The $O^{ - } V_{Al}$ center was studied by the EPR method in undoped α-Al_2_O_3_ exposed to gamma-irradiation^43^ (causes recharging of the existing defects but not the creation of novel ones) as well as in α-Al_2_O_3_:Ti crystals oxidized at high temperatures (enhanced concentration of as-grown aluminium vacancies)^45–48^. For such *V*-type center, the different ratio between the *g* tensor components is valid *g*_y_ ≈ *g*_x_ > *g*_z_, and the maximum value *g* = 2.020 is much lower than *g* = 2.0459 for the $O_{2}^{ - }$ center revealed in the present study. According to Refs.^45^ and ^46^, the hole release from the *V*-type center occurs at 370–530 K, in significantly lower temperature region than the thermal destruction of the $O_{2}^{ - }$ center being discussed. It should be noted that we did not succeed in detecting the EPR signal of the $O^{ - } V_{Al}$ center in our neutron-irradiated α-Al_2_O_3_ crystals. The absence of these *V*-type centers centers or their rather small concentration (lower than 10^16^ cm^-3^) in our samples may be explained by the formation of oxygen interstitials—more deep hole traps that the *V*_*Al*_ (see, also^26^).

It was mentioned already that the pronounced HFS in the EPR lines of the $O_{2}^{ - }$ center is not detected regardless of registration temperature. This fact clearly indicates that the center is composed of mainly oxygen because this element, unlike aluminium, has only negligible amount of nuclei with a nonzero magnetic moment (only for ^17^O isotope with abundance of 0.04%, *I* = 5/2). In general, the presence of HFS due to the $O_{2}^{ - }$ center interaction with Al^3+^ nuclei is not excluded. However, such possible interaction, if any, is a weak one and the HFS could not be resolved in case of a rather wide EPR line (at least 1.5 mT). It is worth noting that the HFS for the $O_{2}^{ - }$ center in SrO (based on an as-grown structural defect) was estimated as about 0.3 mT^35^ and a similar value could be expected in Al_2_O_3_ as well.

**Figure 5:**
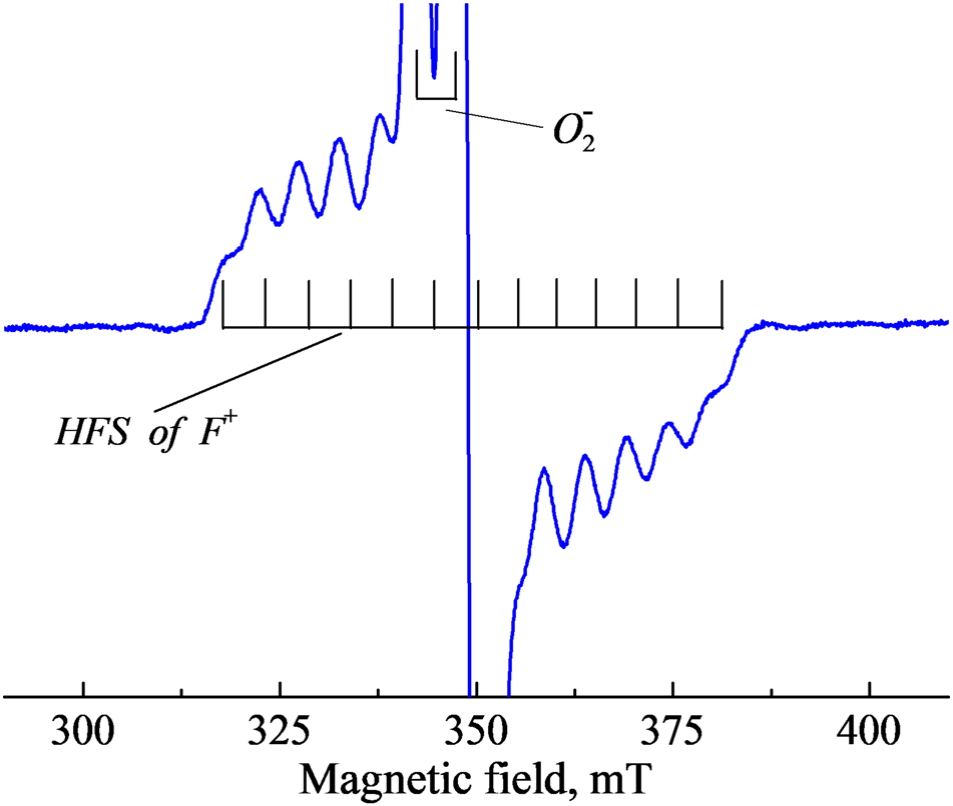
The *F*^+^ center EPR spectrum for a neutron-irradiated Al_2_O_3_ crystal. *P* = 0.6 mW, RT, **B**⊥*c* and **B** || *a* (**B** lies in the (0001) plane).

At the same time, the HFS is clearly observed in the EPR spectrum of the *F*^+^ center, which was revealed in a neutron-irradiated α-Al_2_O_3_ crystal and thoroughly interpreted in Ref.^24^. Fig. 5 demonstrates the *F*^+^ center spectrum for our neutron-irradiated crystal. It comprises 13 isotropic and equidistant lines at **B** = 315–380 mT due to the interaction of the unpaired *s* electron of the *F*^+^ center with two pairs of slightly inequivalent^27^ Al nuclei (nuclear spin *I* = 5/2, natural abundance of 100%, distance to *V*_*O*_ equals 1.86 and 1.97 A, respectively) surrounding the oxygen vacancy $V_{O}$. Note that the central part of the *F*^+^ spectrum is hidden under strong anisotropic lines of the $O_{2}^{ - }$ center and already mentioned unidentified radiation defect with *g* = 2.00 (marked as ♦ in our figures). The concentration of the *F*^+^ centers in our irradiated Al_2_O_3_ crystal (fluence of 6.9×10^18^ *n*/cm^2^) was determined to be close to 1×10^18^ cm^−3^ by means of the Bruker programs. It is important to note that this value practically coincides with the concertation of the $O_{2}^{ - }$ centers determined using the EPR or using the Smakula-Dexter formula for radiation-induced optical absorption of *F*^+^.

Based on the present EPR study, we can state that the radiation-induced $O_{2}^{ - }$ center non-associated with any other structural defect was revealed for the first time in metal oxides. The $O_{2}^{ - }$ molecule presents a kind of an oxygen interstitial that has interacted with a regular oxygen and became stabilized by a trapped hole; this superoxide ion occupies one oxygen site in the (0001) base crystal plane and is oriented along the *a* axis, i.e. the oxygen triangle bisectrix. One can suppose that the *z* axis (*x*, *y* and *z* axes are applied for the *g*-tensor components, i.e. axes of the center) in the (0001) plane may be declined off the *a* direction, wherein a shift by a small angle to one or another side is caused by a minor disorientation of the adjacent oxygen triangles. According to Refs. ^48^ and ^60^, the triangle disorientation value is about 4°. We did not detect the splitting of EPR lines related to such disorientation. However, the large width of the $O_{2}^{ - }$ lines could be determined by a variety of superoxide ions with slightly different orientations (±4° with respect to the *a* axis in the (0001) plane). It was mentioned already that, in general, the broadening of the $O_{2}^{ - }$ EPR lines could be also related to unresolved HFS due to the hole interaction with four nearest-neighboring Al^3+^ or two oxygens forming the $O_{2}^{ - }$ (such HFS will not be detectable for ^16^O isotope). Note that the results on the possible superoxide defect configuration obtained via the density functional theory calculations employing the hybrid B3PW exchange-correlation functional are in line with the present experimental results and will be considered in Sect. 6.

## Thermal annealing of neutron-induced structural defects

Additional information on the origin and microstructure of structural defects could be obtained by studying the radiation damage recovery via consequent thermal annealing of the neutron-irradiated crystal. Note that irreversible annealing of a certain structural defect confirms its “radiation-induced origin”, while reappearance of such centers, after additional irradiation by X-rays at RT, in a totally annealed crystal indicates that we are dealing with some kind of as-grown defects, which serve as effective traps for charge carriers formed by X-rays (such radiation type does not create novel structural defects in metal oxides).

**Figure 6:**
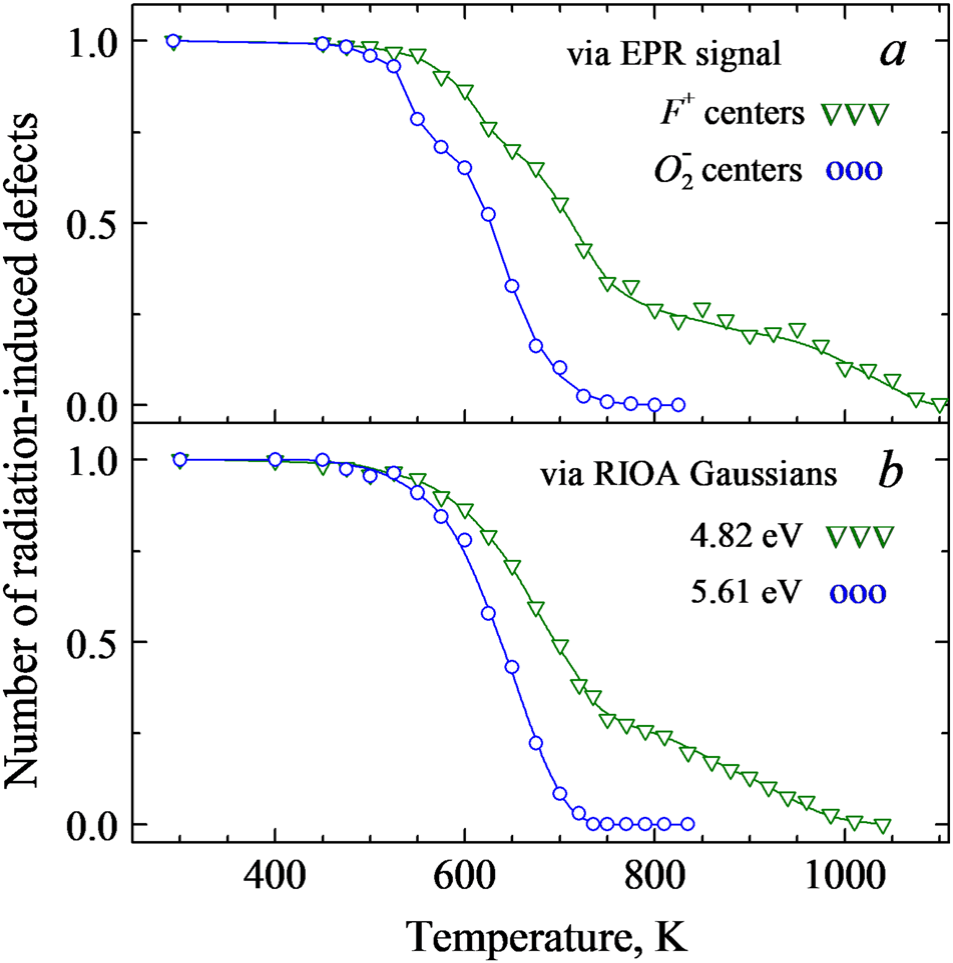
Pulse annealing of the EPR signal of the *F*^+^ and ${O}_{2}^{-}$ centers and Gaussian components of RIOA connected with the *F*^+^ (the band peaked at 4.82 eV) and ${O}_{2}^{-}$ (5.61 eV) centers in an Al_2_O_3_ single crystal irradiated with fast neutrons (6.9×10^18^ cm^-2^, RT). Spectra are normalized, all measurements are performed at RT.

The results of the pulse annealing of the EPR signal of the *F*^+^ and $O_{2}^{ - }$ centers are presented in Fig. 6a. Note that the EPR signals always measured at RT remain permanent within a whole multistage annealing process. The decay of the *F*^+^ EPR signal occurs in two main stages: destruction of two thirds of centers from ~500 to 750 K, and the remaining *F*^+^ (tentatively at least partly associated with some impurity ions, similarly to so-called *F*_A_-type centers in alkali halides) become annealed at 750−1100 K. Because of initially rather wide EPR lines related to the F^+^ center, we did not detect their additional broadening due to disturbance by an addition impurity defect. The annealing of the EPR signal of the $O_{2}^{ - }$ centers has been implemented in a similar way, although both stages with different ratio start at lower temperatures and the annealing is practically completed to 760 K. Both paramagnetic centers are definitely radiation-induced defects. Their thermal annealing is irreversible and a subsequent exposure to X-rays of the completely annealed sample (or preheated up to the end of the first stage of the *F*^+^ annealing, *T*_pr_ = 750 K) does not lead to the reappearance (rise) of these EPR-active defects at RT.

It is generally accepted that the annealing of the *F*-type centers in neutron-irradiated metal oxides is due to the recombination of the becoming mobile oxygen interstitials with still immobile anion vacancies being a part of the *F*^+^ and *F* centers. Accordingly, the data depicted in Fig. 6a illustrate the annealing at 500–750 K of two complementary interstitial-vacancy Frenkel defects created by fast fission neutrons via the universal collision mechanism.

**Figure 7:**
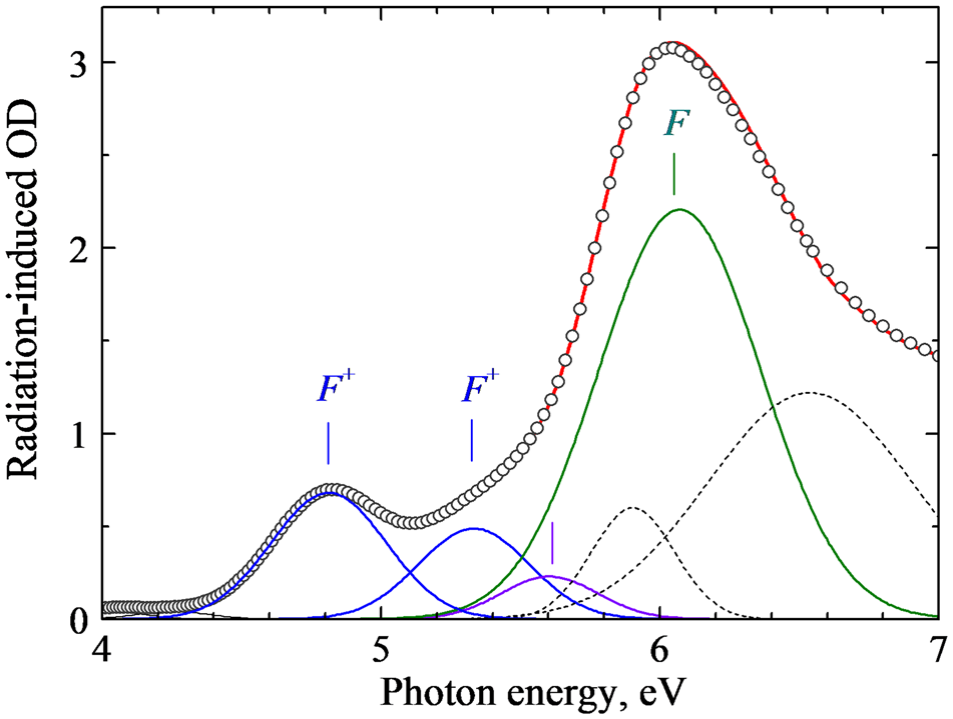
Spectrum of RIOA for an Al_2_O_3_ single crystal irradiated with fast neutrons (6.9×10^18^ cm^-2^, RT, *d* = 0.09 mm) and its decomposition into Gaussians. Symbols represent experimental points measured at RT, sum of components is marked by a solid line.

The parallel study of the thermal annealing of radiation-induced optical absorption was performed for the same crystals (fast neutron fluence of 6.9×10^18^ *n*/cm^2^) and using the same stepwise preheating procedure. Figure 7 shows the RIOA spectrum at 4–7 eV measured for a 0.9-mm thick sample of a neutron-irradiated α-Al_2_O_3_ crystal. This RIOA spectrum is decomposed into Gaussians, part of which is ascribed in the literature to the *F*^+^ (elementary Gaussians peaked at 4.82 and 5.33 eV) and *F* centers (the band maximum at 6.07 eV)^14–16^. A detailed analysis of the thermal annealing of the *F*-type centers and oxygen interstitials measured via the RIOA and EPR (where possible) will be performed and compared with the kinetics simulated in terms of diffusion-controlled reactions within the forthcoming separate paper. Just now we only want to stress that the stepwise annealing of the EPR signal and the corresponding absorption band of the *F*^+^ centers coincide with very high accuracy (see Fig. 6). Therefore, the similar thermal behaviour of the EPR signal of the $O_{2}^{ - }$ centers revealed in this study and the RIOA band with a maximum at 5.6 eV (see the component of decomposition in Fig. 7) allows to attribute this absorption band to the $O_{2}^{ - }$ centers. Note that the annealing of a RIOA band related to the *F* centers, being very similar to that of the *F*^+^ ones, starts at slightly higher temperature.

The main stage of irreversible annealing of the EPR lines (♣) related to some unidentified structural defect takes place to about 760 K, i.e. approximately in the same temperature region as the $O_{2}^{ - }$ centers. According to our preliminary results, the unidentified radiation defect could have a similar microstructure – in particular, the $O_{2}^{ - }$ adjacent to some other lattice defect. Unfortunately, because of rather weak intensity of the (♣) line and its strong overlapping with the EPR signal of single $O_{2}^{ - }$ centers, we did not succeed to measure the angular dependence of this EPR signal, and further studies are needed for strict determination of the origin of the corresponding paramagnetic defect.

It is important to note that the concentration of paramagnetic *F*^+^ and $O_{2}^{ - }$ centers in our neutron-irradiated corundum crystals was estimated to be practically equal, ~1×10^18^ cm^-3^. According to Fig. 6, two thirds of the *F*^+^ centers and all EPR-active $O_{2}^{ - }$ become annealed to about 750 K. Accordingly, the thermal annealing could be considered as a mutual recombination of these Frenkel defects. Low-temperature shift (by ~50-100 K) of the annealing curve for $O_{2}^{ - }$ centers, with respect to that for the *F*^+^ centers, testifies to the firstly decay of a molecular center resulting in the release of an oxygen interstitial (its charge state is unclear yet), which is mobile and recombines with a vacancy-type immobile defect (preferably, with *F*^+^). It is noteworthy that the concentration of the *F* centers created in the α-Al_2_O_3_ crystal by fast fission neutrons is even higher (compare the corresponding Gaussians in Fig. 7), and more oxygen interstitials should exist in the irradiated crystal and serve as a mobile recombination partner for the remaining *F*^+^ and the majority of the *F* centers. A search for others types of oxygen interstitials with nonparamagnetic origin still lies ahead.

## Density functional theory calculations on ${\varvec{O}}_{2}^{ - }$ configurations and properties

Advantage of the DFT calculations is the possibility to model and compare all possible oxidation states for $O_{2}^{OS}$. As discussed in refs. ^40–42^, the *OS* = 2- (*q* = 0) and *OS* = 3- (*q* = -1) lead in corundum to the dumbbell and pseudo-dumbbell configurations, respectively. It is worth mentioning that positive charge states were also considered for interstitial oxygens in ZnO^61^and In_2_O_3_^62^. Several configurations for interstitial oxygens were also identified in these studies. It is worth mentioning that one of the dumbbell configurations (the so-called rotated dumbbell interstitial in ZnO^61^) supports the -1 charge state with larger separation between the two oxygens. The -1 and -2 charge states for the dumbbell configurations were found energetically unfavorable compared to *q* = 0, +1 in In_2_O_3_^62^. Even though the charge state q = -2 for the interstitial oxygen in α-Al_2_O_3_ has been shown in earlier DFT studies^59^ to exist with the smaller formation energy than the -1 charge state, it leads to a diamagnetic state and is, thus, out of the scope of the present study. To the best of our knowledge, no DFT calculations exist for a superoxide defect (q = +1) in α-Al_2_O_3_.

**Figure 8:**
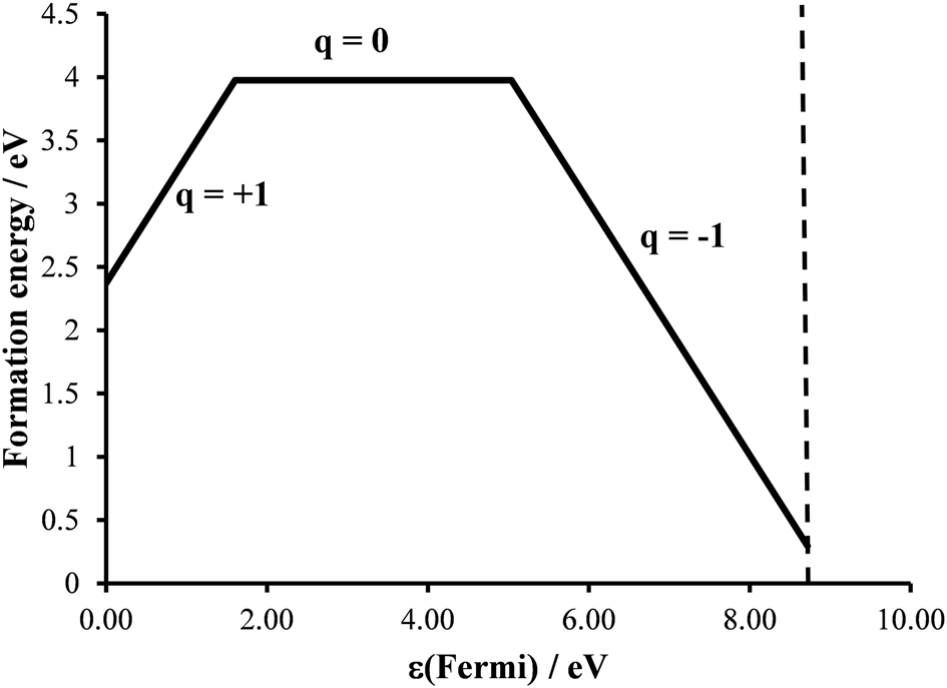
Defect formation energy (${E}_{F}^{q}$, Eq. 1) for paramagnetic ${O}_{2}^{-}$ (*q* = +1, asymmetric dumbbell configuration), and ${O}_{2}^{3-}$(*q* = −1, pseudo-dumbbell configuration) as a function of the Fermi energy (${}_{F}$). ${O}_{2}^{2-}$(q = 0, dumbbell configuration) is given for a consistence. The asymmetric configuration represents the ground state configuration of the present study (see the text for details). The dashed line shows the band gap value.

Figure 8 depicts the defect formation energy ($E_{F}^{q}$) calculated for $O_{2}^{ - }$, $O_{2}^{2 - }$ and $O_{2}^{3 - }$ as a function of the Fermi energy $_{F}$ which varies from the VBM (taken as zero) to the conduction band minimum. It is clearly seen that all three calculated *OS*s have their region of stability. Proximity of $O_{2}^{ - }$ stable region close to the VBM is very much consistent with its hole-type character. It is very reasonable that the two paramagnetic defects, namely $O_{2}^{ - }$ and $O_{2}^{3 - }$, have smaller $E_{F}^{q}$ than the $O_{2}^{2 - }$ one. We, therefore, confirm possible existence of such defects in α-Al_2_O_3_ depending on experimental conditions. In accordance with the experiments above, the superoxide defect is further discussed in detail.

Interestingly, the *OS* = 1− suggests two possible dumbbell configurations: symmetric and asymmetric. Both the configurations are characterized by a short O–O bond length (~1.3 Å vs 1.44 Å in $O_{2}^{2 - }$ and 1.86 Å in $O_{2}^{3 - }$) but differ in distribution (equal/unequal) of the atomic charges and oxygen magnetic moments (Table 2). Notice that the calculated bond length of $O_{2}^{ - }$ is very much consistent with the experimental literature data on superoxide compounds^58^ and calculated isolated superoxide ion (see, ^63^ and references therein). Notice that the existence of two configurations is not so surprising: as was discussed^63^, the inversion symmetry of $O_{2}^{ - }$ is often lost in alkali metal superoxides, MeO_2_, due to low crystalline symmetry. The formation energy difference between the two configurations is of the order of 0.2 eV in the favor of the asymmetric configuration being, thus, a ground state one (see Table 2).

**Table 2:** Basic properties of ${O}_{2}^{-}$ in two configurations. *ΔE* the total energy difference between the two configurations, *Q* the atomic charge of each oxygen atom in the dumbbell calculated within the Mulliken analysis, *M* the magnetic moment of each oxygen atom, *D* the bond length, OVPOP the overlap population between the two oxygens, and Δ*E*_g_ the gap between the valence band maximum and defect band.

Configuration of ${O}_{2}^{-}$	*ΔE*, eV	*D*, Å	*Q*, *e*	*M*, *µ*_*B*_	OVPOP^*^, e	Δ*E*_g_, eV
Symmetric	0.2	1.32	2 × −0.36	2 × 0.42	−0.08	2.19
Asymmetric	0.0	1.34	−0.19 −0.52	0.60 0.14	-0.02	2.51

^*^ The values were multiplied by a factor of 2 in accordance with^55^.

**Figure 9:**
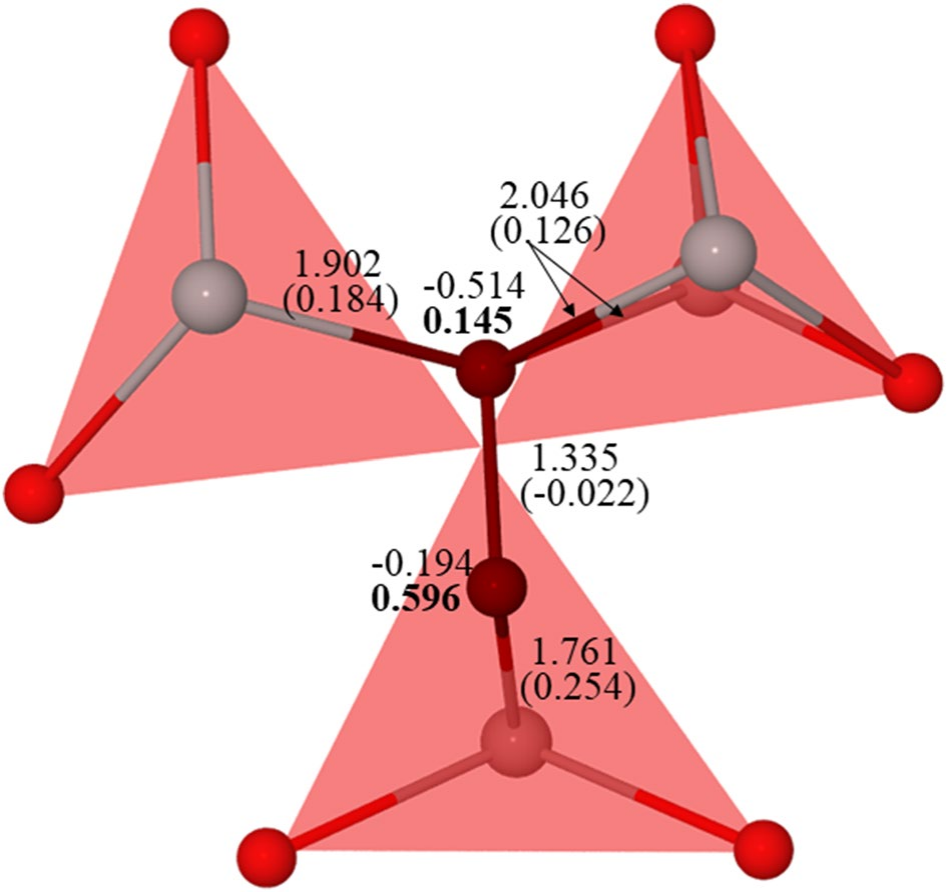
The lattice relaxation pattern around the superoxide defect (${O}_{2}^{-}$) in α-Al_2_O_3_ (top view for the asymmetric dumbbell configuration). The two oxygens of ${O}_{2}^{-}$ are indicated in dark red color. Red: O, grey: Al. The numbers for the two oxygens of ${O}_{2}^{-}$ are magnetic moments in µ_B_ (in bold) and atomic charges in e. The numbers for the Al-O and O–O bonds are overlap (bond) population (in parenthesis), in e, and bond lengths, in Å.

Fig. 9 depicts the lattice relaxation pattern around the $O_{2}^{ - }$ center in the asymmetric configuration. Note that the six oxygen ions in perfect α-Al_2_O_3_ are subdivided into two groups with the Al-O distances 1.86 and 1.97 Å if calculated with the B3PW functional. In the presence of interstitial oxygen the Al-O distances vary from 1.76 to 2.05 Å meaning a quite asymmetric structural relaxation around $O_{2}^{ - }$. The shortest distance of 1.76 Å is between the interstitial oxygen and closest Al^3+^ ion. The bond between these two ions is characterized by the overlap (bond) population of +0.25 *e*. The interstitial oxygen has only one Al^3+^ ion in the vicinity whereas the other neighbors are oxygens. The other (host) oxygen ion of $O_{2}^{ - }$ has the distances 1.90–2.05 Å with three Al^3+^ ions (only two Al^3+^ ions are shown in Fig. 9). Typically the Al-O bond in this case is also characterized by a positive value of overlap population varying from +0.13 to +0.18 *e*.

The atomic charges (*Q*) and magnetic moments (*M*) of two oxygens of $O_{2}^{ - }$ are quite different in the asymmetric configuration. Notice that the interstitial oxygen has larger *M* and smaller *Q* than another oxygen. Besides, the total *Q* and *M* of asymmetric configuration are smaller than in the symmetric case because there is a significant effect of electron delocalization (discussed below). For example, the magnetic moment of one more oxygen at the distance 2.19 Å from interstitial oxygen is 0.16 *µ*_*B*_ in the asymmetric configuration.

However, the two configurations have also similarities. The total *Q* of $O_{2}^{ - }$ (about −0.7 *e*) is different from that in the dumbbell ($O_{2}^{2 - }$) configuration and *Q* of other oxygens in the supercell for both the asymmetric and symmetric configurations. The value of *Q* on oxygen in the perfect crystal due to covalency effects in Al-O chemical bonds differ from the formal −2 *e* and is close to -1 *e* but the total *Q* of $O_{2}^{2 - }$ is only −1.20 *e*. The total *M* (0.84 and 0.74 *µ*_*B*_ in the symmetric and asymmetric configuration, respectively, vs 0.98 *µ*_*B*_ in $O_{2}^{3 - }$) of $O_{2}^{ - }$ in the two configurations corresponds to the experimentally suggested *S* = 1/2. Interestingly, the overlap (bond) population between the two oxygens in $O_{2}^{ - }$ is close to zero, in contrast to almost -0.14 *e* in the dumbbell ($O_{2}^{2 - }$) configuration. Obviously, it is related to different number of electrons occupying the antibonding π^*^2*p*—molecular orbital in the $O_{2}^{ - }$ and $O_{2}^{2 - }$ dumbbells. For a comparison, the Mulliken bond population in a free O_2_ molecule is 0.22 *e*.

In supplementary information we show a fragment of supercell containing the superoxide defect in the two configurations, respectively. In the case of symmetric configuration (Supplementary Fig. S1), the defect lays along the oxygen triangles edges in the direction $\left[ {1\overline{1}00} \right]$. It is inclined to and rotated in the oxygen triangles plane. However, the asymmetric configuration suggests a more sophisticated result when compared with the discussion given in Sect. 4. That is, the defect is oriented along $\left[ {1\overline{2}10} \right]$ but inclined by approx. 17° to the oxygen triangle plane (see Supplementary Fig. S2). In contrast to symmetric configuration, its center is shifted which is in line with asymmetric properties.

**Figure 10:**
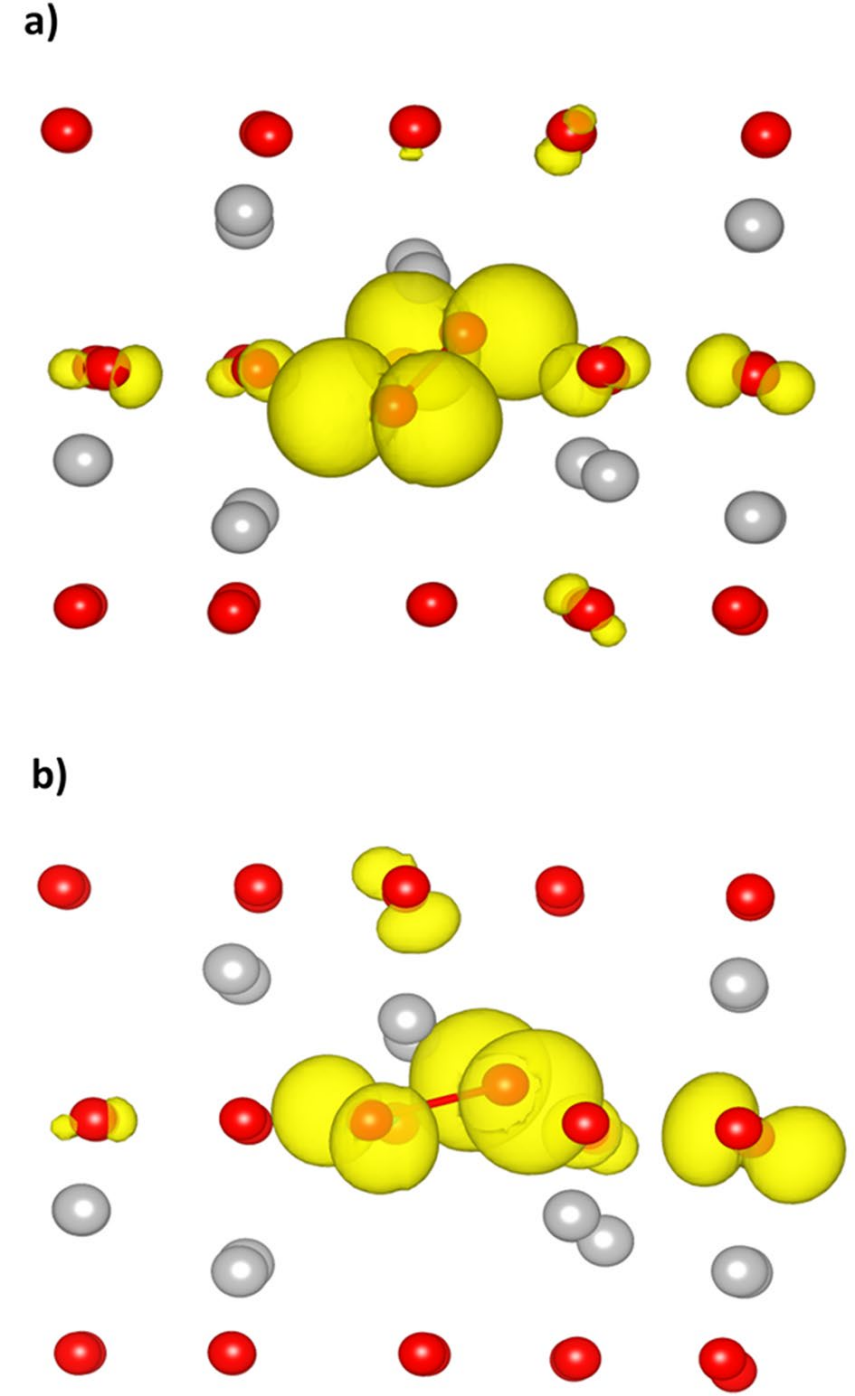
The 3D spin density distribution around and within ${O}_{2}^{-}$ for (**a**) symmetric and (**b**) asymmetric dumbbell configuration. The bond in ${O}_{2}^{-}$ is also shown for clearance. Red balls: oxygens, grey balls: Al, yellow: lobed shaped 2*p* orbitals. The view is chosen to better show the distribution.

As discussed above, both the configurations affect the electron delocalization well seen in Fig. 10, which shows a 3D spin density distribution for the two configurations. There are two lobed shaped O2*p* orbitals in both cases around the two oxygens in $O_{2}^{ - }$ aligned in the same direction. It reflects a major part of the spin density localized on two oxygens in $O_{2}^{ - }$ and an additional spin density on other oxygens surrounding the superoxide defect. The asymmetric configuration has a decreased spin density on the host oxygen in $O_{2}^{ - }$ but increased spin density on the neighbor host oxygen in comparison with the symmetric case.

## Concluding remarks

An EPR study of a novel structural defect in fast-neutron-irradiated α-Al_2_O_3_ single crystals has been performed. According to the angular dependences of the EPR line positions at the rotation of the magnetic field in different crystallographic planes as well as the determined *g* tensor components, the detected hole-type center is identified as the single superoxide ion—the $O_{2}^{ - }$ molecule that occupies one oxygen site in the (0001) crystal plane and is oriented along the *a* axis, i.e. the oxygen triangle bisectrix. The $O_{2}^{ - }$ center consists of the regular and interstitial oxygens being stabilized by a trapped hole. Thus, for the first time, radiation-induced superoxide ion without any other structural imperfection/defect in its immediate vicinity has been observed in metal oxides. The thermal annealing of the paramagnetic $O_{2}^{ - }$. centers occurs at 500-750 K via their dissociation and subsequent recombination of the becoming mobile oxygen interstitials, probably as ions in the formal charge state −1, with still immobile single *F*-type centers (mostly with the EPR-active *F*^+^ centers).

We also modeled single $O_{2}^{ - }$ centers using the DFT calculations with the hybrid BW exchange-correlation functional. The callated properties include the atomic structure, lattice relaxation, formation energies, atomic charges, magnetic moments, and spin density distribution. Such properties are not accessible to the experiments and, thus, the DFT calculations represent a good complement to them. The two dumbbell configurations for the $O_{2}^{ - }$. center are suggested on the basis of DFT calculations. The ground state configuration with *S* = 1/2 and hole-type character of the novel hole defect is perfectly consistent with the presented EPR measurements.

## Supplementary information

Supplementary information
