# Supplementary material for: Atomic, electronic and magnetic structure of an oxygen interstitial in neutron-irradiated Al2O3 single crystals
Source: Sci Rep. 2020 Sep 28;10:15852. doi: 10.1038/s41598-020-72958-9 (PMC7522295; doi:10.1038/s41598-020-72958-9)
Supplement: Supplementary file 1 — Supplementary information [file 41598_2020_72958_MOESM1_ESM.docx]

**Supplementary information**

**Atomic, electronic and magnetic structure of an oxygen interstitial in neutron-irradiated Al_2_O_3_ single crystals**

V. Seeman^a^, A. Lushchik^a^[[1]](#footnote-1)^^, E. Shablonin^a^ , G. Prieditis^a^_,_ D. Gryaznov^b2^, A. Platonenko^b^, E.A. Kotomin^b,c^, A.I. Popov^a,b,d^

*^a^Institute of Physics, University of Tartu, W. Ostwald Str. 1, Tartu 50411, Estonia*

*^b^Institute of Solid State Physics, University of Latvia, Kengaraga 8, Riga LV-1063, Latvia*

*^c^Max Planck Institute for Solid State Research, Heisenbergstr. 1, Stuttgart D-70569, Germany*

*^d^Institute Laue Langevin, 6 rue Jule Horovitz, Grenoble 38042, France*

III

II

I


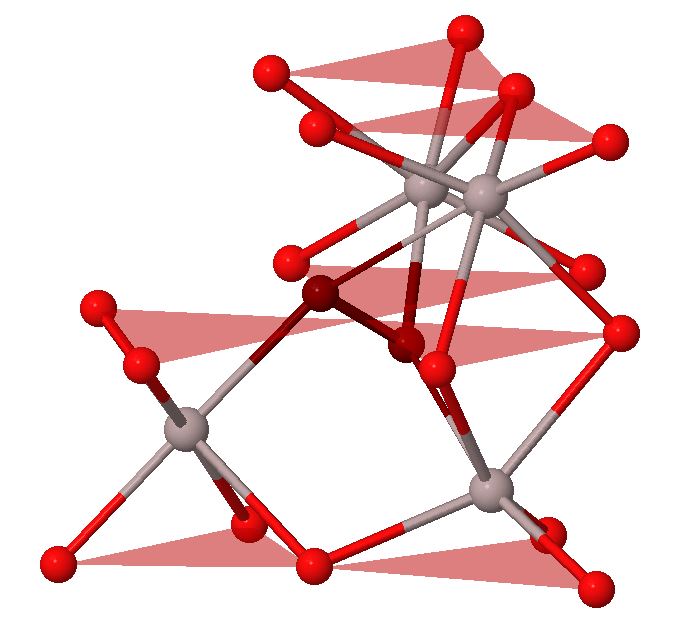


**a)**

3

1

2


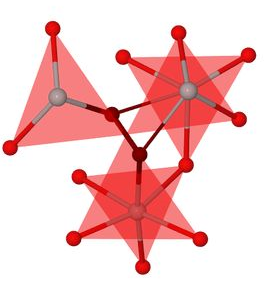


**b)**

3

1

2

[1$\overline{2}$10]

[1$\overline{1}$00]

[0001]

Figure S1. A fragment of supercell containing the superoxide ($O_{2}^{-}$) defect (symmetric configuration): a) side view b) top view. Red balls: oxygens, grey balls: Al. Three layers of oxygen triangles (I, II, III) are shown. The superoxide ($O_{2}^{-}$ ) defect lies in the layer II, its oxygens are indicated in dark red color. Numbers of oxygen triangles (1, 2, 3) in the layer II are shown for convenience. Only layers II and III are shown in b).

Figure S2. A fragment of supercell containing the superoxide ($O_{2}^{-}$) defect (asymmetric configuration): a) side view b) top view. Red balls: oxygens, grey balls: Al. Three layers of oxygen triangles (I, II, III) are shown. The superoxide ($O_{2}^{-}$) defect lies in the layer II, its oxygens are indicated in dark red color. Numbers of oxygen triangles (1, 2, 3) in the layer II are shown for convenience. Only layers II and III are shown in b).


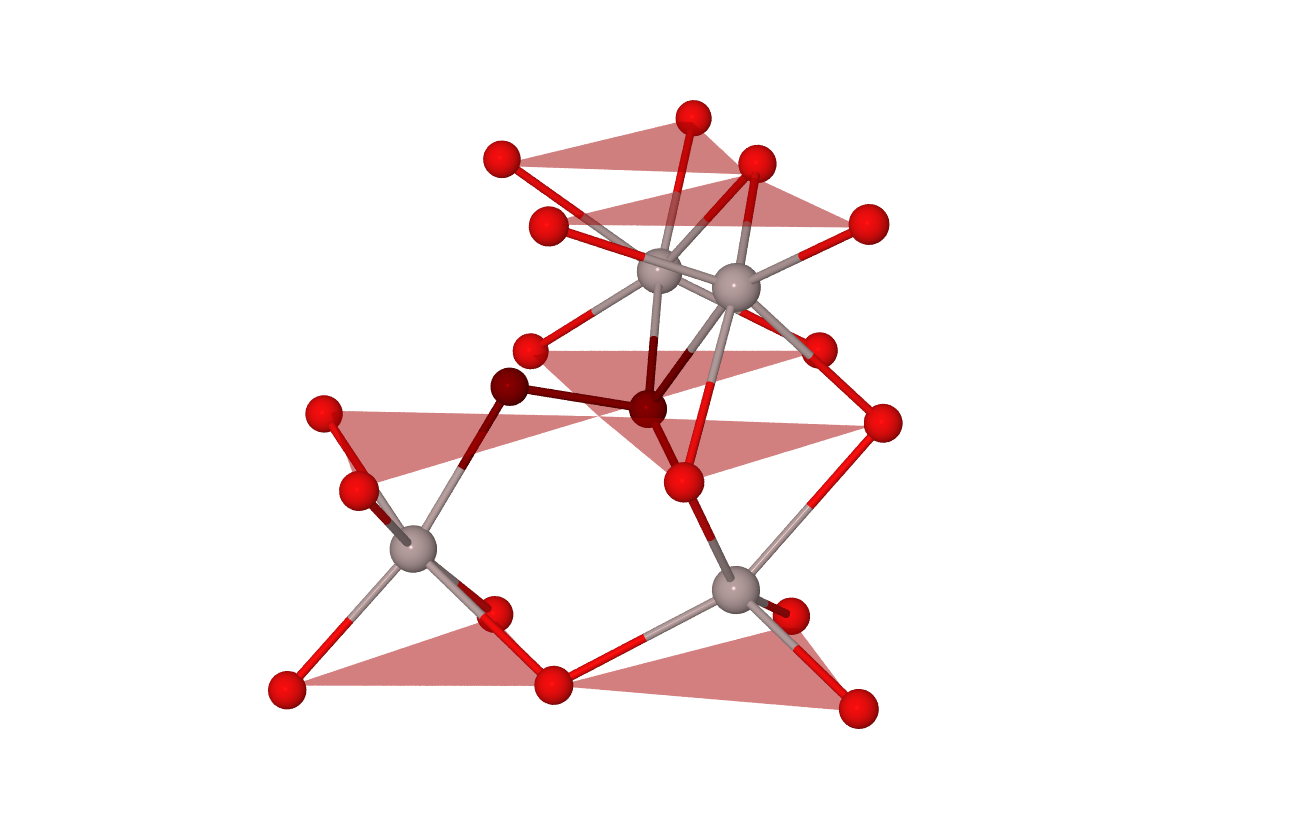


2


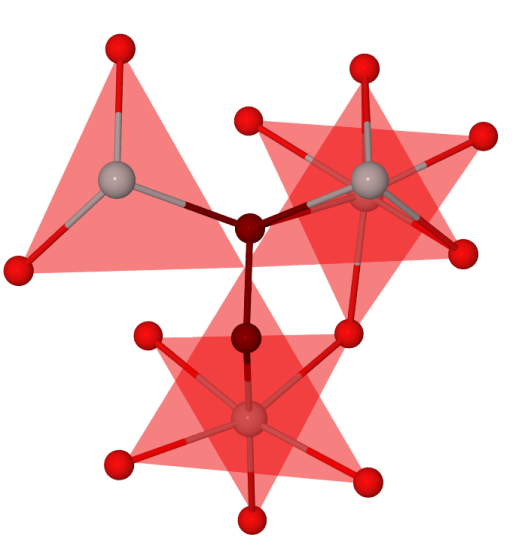


[1$\overline{2}$10]

[1$\overline{1}$00]

[0001]

1

3

III

II

I

**b)**

**a)**

1. [↑](#footnote-ref-1)
